# Primary hyperparathyroidism in a domestic shorthair cat following I^131^ radioiodine therapy

**DOI:** 10.1111/jsap.13854

**Published:** 2025-04-01

**Authors:** E. Ruane, P. Odatzoglou, H. Wong, A. Hayes

**Affiliations:** ^1^ Department of Veterinary Medicine University of Cambridge Cambridge UK

## Abstract

A 12‐year‐old male neutered domestic shorthaired cat presented for further assessment of a cervical mass, having undergone radioiodine therapy for treatment of hyperthyroidism 2 years prior to presentation. Initial cytological diagnosis of the mass was supportive of a carcinoma and laboratory results were consistent with primary hyperparathyroidism. Thoracic radiographs and magnetic resonance imaging were performed of the neck prior to surgical removal of the mass. Histopathology and immunohistochemical characteristics were supportive of a parathyroid tumour. Primary hyperparathyroidism and the associated hypercalcaemia fully resolved following surgery. In human medicine, there is awareness of an association between radioactive iodine therapy and development of primary hyperparathyroidism. Based on our literature search, this sequence of pathologies has not been reported in cats. This report documents primary hyperparathyroidism in a cat following radioiodine therapy.

## INTRODUCTION

Hypercalcaemia is defined as an increase in serum calcium level above the upper limit of normal for a given reference value (Endres, 2012). Parathyroid hormone (PTH) is the principal hormone involved in the regulation of blood calcium concentration, synthesised in the parathyroid glands. Cats have two parathyroid glands associated with each lobe of the thyroid gland. PTH increases serum calcium concentrations and decreases serum phosphate concentrations via three main target organs: bone, kidneys and gastrointestinal tract (Finch, 2016).

Idiopathic hypercalcaemia is considered the most common diagnosis in hypercalcaemic cats (Finch, 2016). In two recent studies, only 2.1% (Broughton et al., 2023) and 5.6% (Savary et al., 2000) of hypercalcaemic cats were diagnosed with primary hyperparathyroidism (pHPT). Most cats with pHPT are affected by single parathyroid gland adenomas (Parker et al., 2015; Singh et al., 2019). Fewer are diagnosed with primary parathyroid hyperplasia, carcinomas or cystadenomas (Bonczynski, 2007; Kallet et al., 1991; Singh et al., 2019).

Since the first case report by Rosen and colleagues in 1975, multiple studies have documented an association between external radiation and pHPT in people (Bobanga et al., 2023). Radioactive iodine (RAI) is used in human medicine to treat benign and malignant thyroid neoplasms, and hyperplastic diseases such as Graves' disease (Law et al., 2021), and dose protocols vary depending on the specific conditions being treated. RAI is thought to be a risk factor for pHPT, although a dose‐dependent correlation between RAI exposure and incidence of pHPT has not been determined. The authors postulate that although the parathyroid glands do not concentrate iodine, their proximity to the thyroid gland may result in exposure to radiation and subsequent development of pHPT (Bobanga et al., 2023).

A search of the Medline (PubMed), Google Scholar and ScienceDirect databases with search terms “radioiodine OR ‘radioactive iodine’ OR ‘Iodine‐131’ AND cats OR feline AND hyperparathyroidism, primary [Mesh] OR hypercalcaemia” (authors'' last search on April 8, 2024) was performed. No reports of pHPT in a cat following radioiodine therapy were found. Here we present a case of pHPT in a cat following radioiodine therapy.

## CASE PRESENTATION

**FIG 1:**
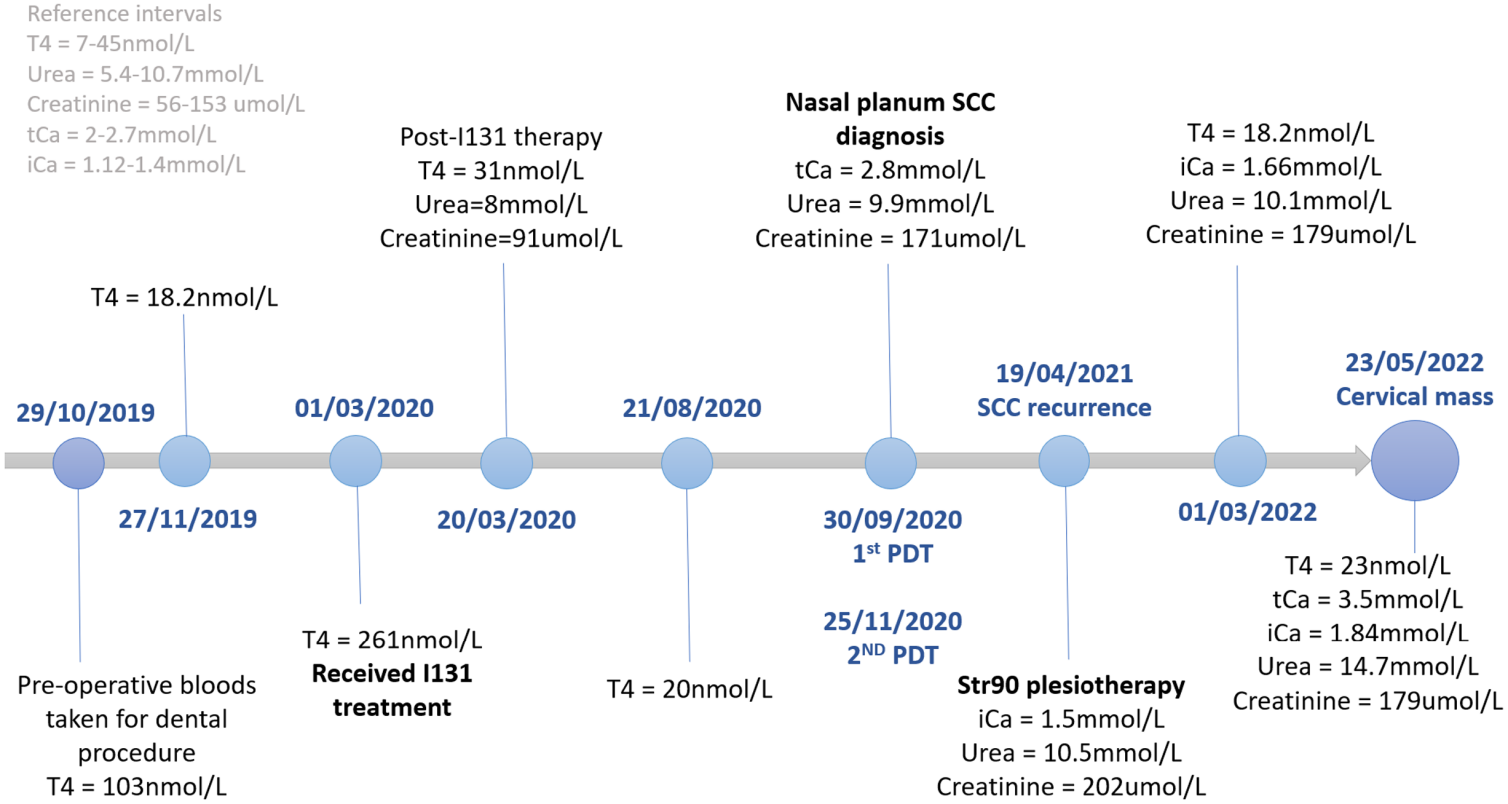
Timeline illustrating clinical history of the case prior to presentation for cervical mass (PDT Photodynamic therapy, SCC Squamous cell carcinoma).

A 12‐year‐old male neutered domestic shorthaired cat (bodyweight 4 kg Body Condition Score (BCS) 5/9) was referred for assessment of a cervical mass. This had been cytologically diagnosed by a board‐certified clinical pathologist as a possible carcinoma. One year prior, the cat underwent treatment with photodynamic therapy for a nasal planum squamous cell carcinoma at the authors' institution. A partial response was seen and follow‐up treatment with strontium 90 plesiotherapy resulted in complete clinical response. Previous clinical history (Fig 1) included radioiodine treatment for management of hyperthyroidism at a separate referral institution 2 years prior to presentation.

Hypercalcaemia was first documented at presentation for strontium plesiotherapy, total calcium (3.1 mmol/L ref, 2 to 2.7) and ionised calcium (1.5 mmol/L ref, 1.12 to 1.43), and at follow‐up 2 months later. Due to the lack of clinical signs of hypercalcaemia, the owners declined further investigations.

Other relevant clinical history included subclinical hypertrophic cardiomyopathy and IRIS Stage II chronic kidney disease (CKD). A phosphate‐restricted diet was advised but the owners were non‐compliant. The patient was reported to be polyuric and polydipsic on presentation but otherwise was well. Clinical examination revealed a firm, mobile, non‐painful mass measuring 3.2 to 3.5 cm on the left ventral aspect of the neck and a Grade III/VI right‐sided heart murmur. The rest of clinical examination was within normal limits.

Complete blood count at presentation was unremarkable. Serum biochemistry revealed azotaemia: urea (14.7 mmol/L ref, 5.4 to 10.7), creatinine (179 μmol/L ref, 56 to 153) and increased total (3.5 mmol/L ref, 2 to 2.7) and ionised calcium (1.84 mmol/L ref, 1.12 to 1.4 ). Phosphate concentration was low to normal (1.1 mmol/L ref, 0.9 to 2.1). Serum PTH, measured using a chemiluminescent assay, was elevated (73 pg/mL ref, 0 to 20); PTH‐related peptide (PTHrp) and total thyroxine (T4) concentration were normal. Thyroid‐stimulating hormone was not measured. Urine obtained via cystocentesis showed isosthenuria (USG 1.016) with a normal urine protein–creatinine ratio. Systolic blood pressure measured indirectly using Doppler ultrasound was normal.

**FIG 2:**
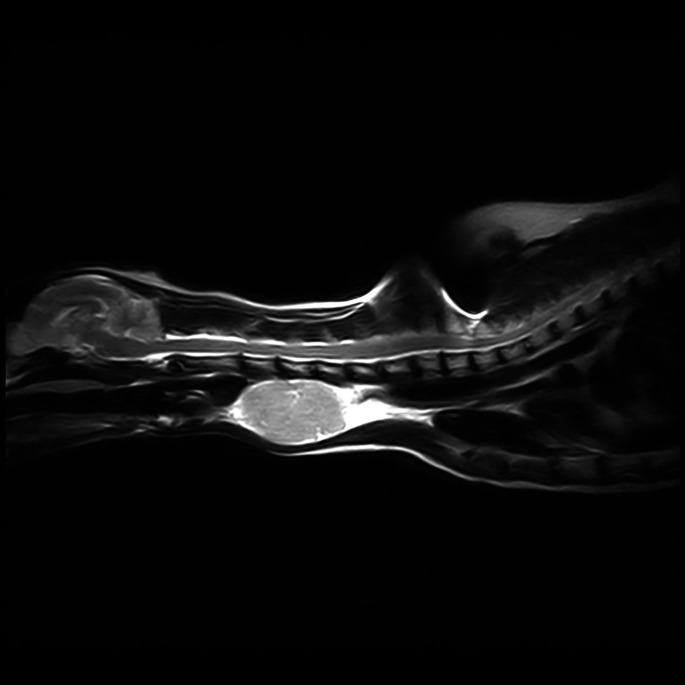
MRI of the Neck T2w sagittal and STIR dorsal sections, respectively. At the level of the mid ventral cervical spine, there is a large heterogeneously T2w/STIR hyperintense mass lesion. The mass is lateral to the trachea, which is displaced to the right. At the centre of the mass, there are multifocal regions of T2w markedly hyperintense material. Reading MRI of the neck 2a) T2W saggital and 2b) STIR dorsal sections respectively.

**FIG 3:**
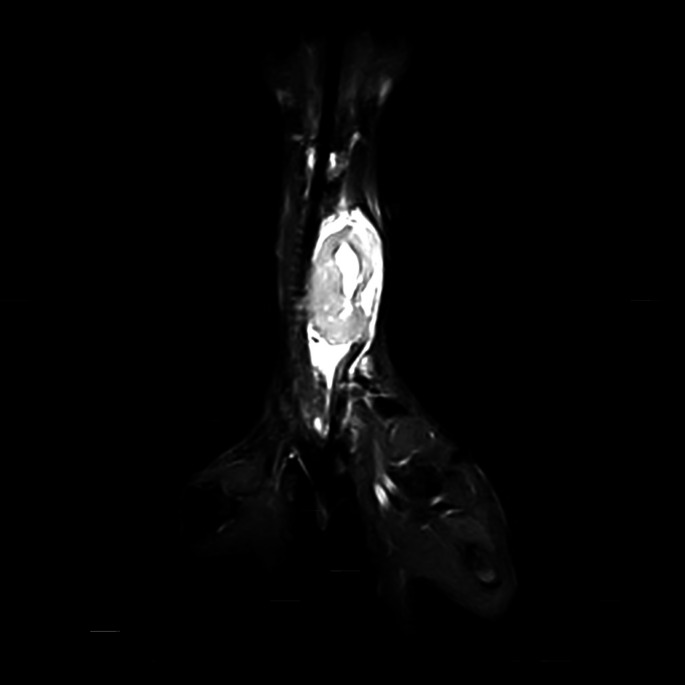
MRI of the Neck T2w sagittal and STIR dorsal sections, respectively. At the level of the mid ventral cervical spine, there is a large heterogeneously T2w/STIR hyperintense mass lesion. The mass is lateral to the trachea, which is displaced to the right. At the centre of the mass, there are multifocal regions of T2w markedly hyperintense material.

Thoracic radiographs showed chronic bronchial airway disease and chronic healed rib fractures, with no gross evidence of metastatic disease. MRI demonstrated a large heterogeneously T2w/ Short tau inversion recovery (STIR) hyperintense and T1w mildly hyperintense mass lesion at the level of the mid ventral cervical spine (Figs 2 and 3). The left parathyroids were not visible. The right thyroid and parathyroids were within normal limits. It was unclear whether the mass was thyroid or parathyroid in origin; however, the elevation in PTH raised suspicion of the latter. Based on the imaging findings and suspicion of parathyroid neoplasia, surgical removal was elected.

**FIG 4:**
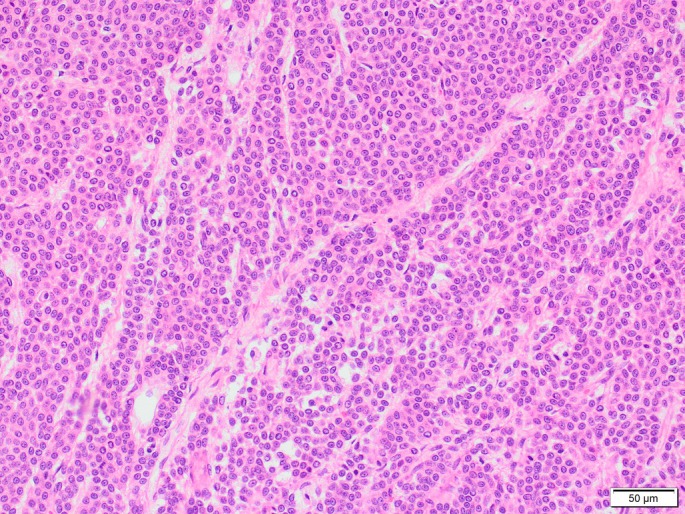
Cervical mass, H&E ×200 magnification. Neoplastic cells form palisading trabeculae and rare acini. Four mitoses per ten HPF (2.37 mm^2^).

Histopathology was suggestive of an epithelial or neuroendocrine neoplasm, of likely thyroid or parathyroid origin (Fig 4). The surgical margins were deemed histologically tumour free, with the mass distinct from surrounding tissue with no evidence of local invasion.

**FIG 5:**
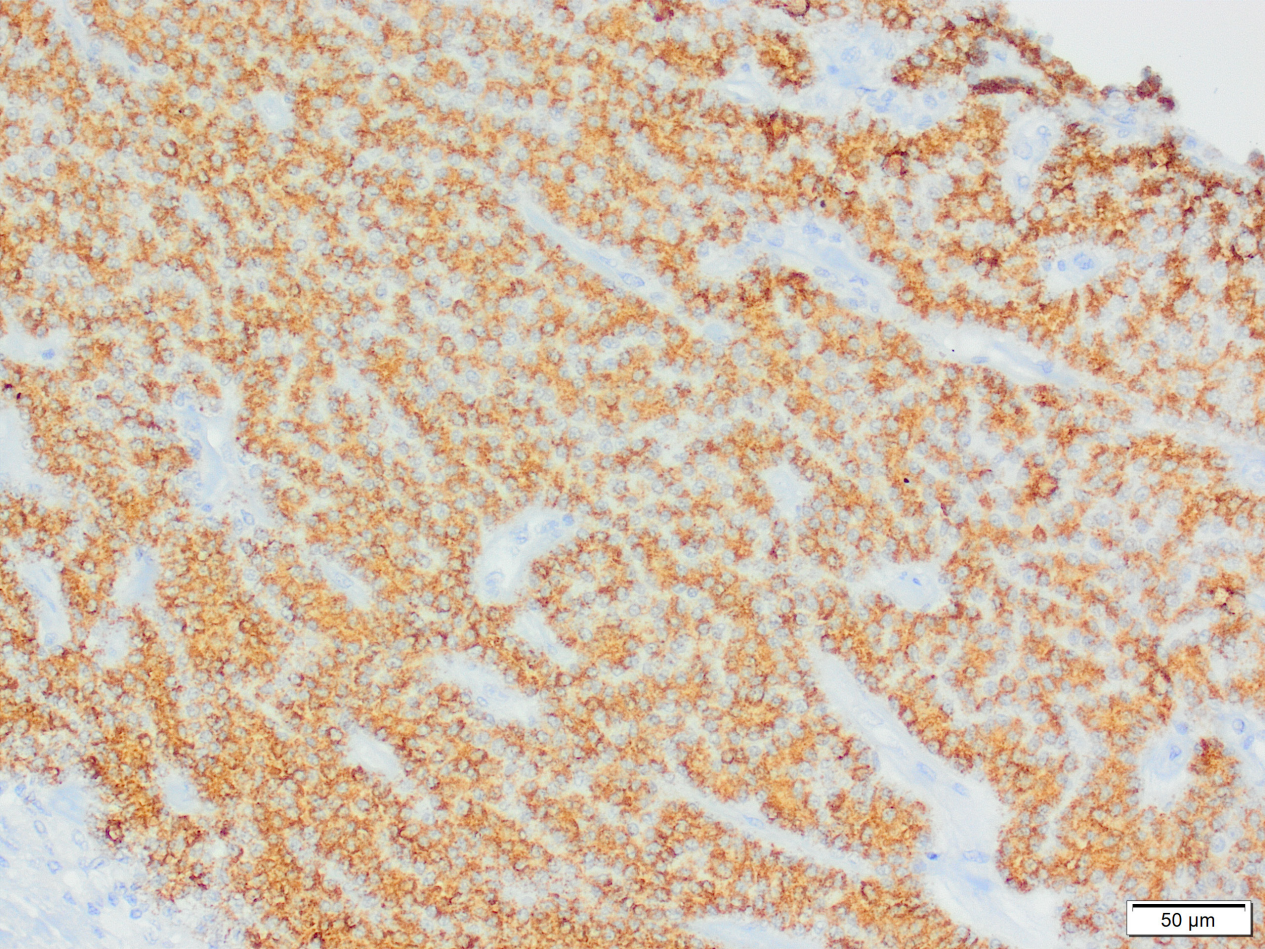
Cervical mass, chromogranin A immunohistochemistry ×200 magnification. Neoplastic cells exhibit granular cytoplasmic immunoreactivity to chromogranin. Neoplastic cells form thin trabeculae and rare acini.

Immunohistochemical staining for cytokeratin, CD18, vimentin, S100, thyroglobulin, chromogranin A and synaptophysin was performed on the tissue sections to further characterise the neoplasm. The sections showed uniform positive immunoreactivity for S100 and vimentin, and moderate to strong positive immunoreactivity to chromogranin A in 80% of neoplastic cells (Fig 5). Sparse numbers of cells within the proliferative cell population exhibited positive cytokeratin immunoreactivity. There was no reactivity for thyroglobulin, CD18 and synaptophysin. Parathyroid tumours are classically strongly cytokeratin and chromogranin A immunoreactive with absent‐to‐variable vimentin immunoreactivity. The vimentin immunoreactivity with minimal cytokeratin immunoreactivity raised the potential for a paraganglioma. However, in this case, the histological architecture of palisading polygonal cells and rare acini, together with the documented PTH changes, was most consistent with a tumour of parathyroid origin with atypical molecular marker expression.

The cat was hospitalised for 4 days post‐operatively. Treatment with 0.05 mL PO Alfacalcidol (2 μg/mL solution) twice a day was initiated in the immediate post‐operative period to prevent clinical signs of hypocalcaemia. Ionised calcium reduced from 1.8 mmol/L preoperatively to 1.35 mmol/L (ref, 1.12 to 1.4) at discharge.

Clinical checks were made fortnightly by the referring veterinary surgeon, with the ionised calcium measurements detailed in Table 1. Alfacalcidol dose was tapered and withdrawn when consistent normocalcaemia was documented 152 days after surgery. The patient remains normocalcaemic and azotaemic, consistent with IRIS Stage II CKD, at the time of writing, 17 months after surgery.

**Table 1:** Ionised calcium results preoperatively and post‐operatively, and relevant changes to management of the case (iCa = ionised calcium)

Date	iCa (reference interval, 1.12 to 1.4 mmol/L)	Comments
May 23, 2022	1.85	Preop
May 29, 2022	1.3	Alfacalcidol (2 mcg/mL) started 0.05 mL twice a day
June 7, 2022	1.27	Alfacalcidol continued same dose
June 14, 2022	1.49	Reduced to 0.05 mL once a day
July 2, 2022	1.33	Continued same dose
July 22, 2022	1.23	Continued same dose
August 12, 2022	1.24	Reduced dose to 0.05 mL every other day
September 3, 2022	1.3	Reduced dose to 0.05 mL every 72 hours
September 29, 2022	1.29	Stopped Alfacalcidol
October 25, 2022	1.25	Stopped iCa monitoring

## DISCUSSION

The present case report documents the clinical features and subsequent treatment of pHPT in a cat following radioiodine therapy.

Radioiodine is generally considered the treatment of choice for hyperthyroidism in cats due to its safety and efficacy (Peterson, 2006; Peterson & Becker, 1995). Aside from prolonged hospitalisation, costs, and the risk of iatrogenic hypothyroidism, there are few drawbacks to this therapeutic option (Peterson & Becker, 1995).

Feline pHPT is reported infrequently in the literature (Kallet et al., 1991; Savary et al., 2000; Singh et al., 2019). Affected cats are typically middle aged to older, and clinical signs include vomiting, polyuria, polydipsia, weight loss, and a palpable cervical mass (Parker et al., 2015). A normal to high PTH concentration in the face of hypercalcaemia is considered diagnostic for pHPT (Barber, 2004). The present case documents elevated ionised calcium, within reference range phosphate concentration, elevated PTH and normal PTHrp. The presence of a mass lesion in the region of the parathyroid glands, imaging findings and complete resolution of the hypercalcaemia following surgical resection of the mass, further support this diagnosis.

Multiple studies have documented I^131^ RAI therapy to be a risk factor for pHPT in people (Bondeson et al., 1989; Colaço et al., 2007; Esselstyn et al., 1982; Gomez & Shulman, 2014). A prevalence of 2.5% (Bobanga et al., 2023) and 6% (Law et al., 2021) is reported in two studies of RAI‐treated people, where RAI was predominantly utilised as treatment for Graves' disease or toxic nodular goitres. This is higher than the overall prevalence of pHPT in the general population; 0.23% and 0.085% for women and men, respectively, in the US (Yeh et al., 2013). Other reports, however, have not demonstrated a significant association between RAI exposure for thyrotoxicosis and pHPT in people (Fjälling et al., 1983; Rasmuson & Tavelin, 2006). Explanations for this disparity may be the age at exposure to RAI, dose of RAI administered or length of follow‐up (Campennì et al., 2023). RAI dose differs in human medicine by condition. Higher doses (370 to 1110 MBq) are administered for toxic and non‐toxic nodular goitres, compared to Graves' disease (370 to 555 MBq) (Campennì et al., 2023). In veterinary medicine, the dose of RAI employed to treat hyperthyroidism is determined by individualised protocols or the use of fixed standard doses (Lucy et al., 2017; Peterson & Becker, 1995; Vagney et al., 2018). Fixed, standard dosing of RAI in cats in the UK may range from 53 to 300 MBq for thyroid adenomas and 900 to 1100 MBq for thyroid carcinoma depending on the institution. Unfortunately, any direct comparison between RAI dose could not be made in the present case as the dose is not known due to closure of the institution.

Based on our literature search, pHPT has not been previously reported in cats receiving RAI, despite large cohort studies evaluating outcomes in these patients (Slater et al., 2001). The findings reported here document the development of hyperparathyroidism in a cat following RAI therapy.

## Author contributions

**E. Ruane:** Writing – original draft (lead). **P. Odatzoglou:** Investigation (lead); methodology (equal); writing – review and editing (equal). **H. Wong:** Investigation (equal); methodology (equal); resources (equal); supervision (supporting); writing – review and editing (supporting). **A. Hayes:** Conceptualization (lead); investigation (lead); methodology (lead); supervision (lead); writing – review and editing (supporting).

## Conflict of interest

The authors declare that they have no conflicts of interest.

## Data Availability

Data sharing is not applicable to this case report as no data sets were generated. All relevant findings are presented in the article.
